# A redox‐active HybG‐HypD scaffold complex is required for optimal ATPase activity during [NiFe]‐hydrogenase maturation in *Escherichia coli*

**DOI:** 10.1002/2211-5463.13546

**Published:** 2023-01-20

**Authors:** Alexander Haase, R. Gary Sawers

**Affiliations:** ^1^ Institute for Biology/Microbiology Martin‐Luther University Halle‐Wittenberg Germany

**Keywords:** [NiFe]‐hydrogenase maturation, ATPase, cysteine residues, HybG‐HypD scaffold, thioredoxin fold

## Abstract

Four Hyp proteins build a scaffold complex upon which the Fe(CN)_2_CO group of the [NiFe]‐cofactor of hydrogenases (Hyd) is made. Two of these Hyp proteins, the redox‐active, [4Fe‐4S]‐containing HypD protein and the HypC chaperone, form the basis of this scaffold complex. Two different scaffold complexes exist in *Escherichia coli*, HypCD, and the paralogous HybG‐HypD complex, both of which exhibit ATPase activity. Apart from a Rossmann fold, there is no obvious ATP‐binding site in HypD. The aim of this study, therefore, was to identify amino acid motifs in HypD that are required for the ATPase activity of the HybG‐HypD scaffold complex. Amino acid‐exchange variants in three conserved motifs within HypD were generated. Variants in which individual cysteine residues coordinating the iron–sulfur ([4Fe‐4S]) cluster were exchanged abolished Hyd enzyme activity and reduced ATPase activity but also destabilized the complex. Two conserved cysteine residues, C69 and C72, form part of HypD's Rossmann fold and play a role in HypD's thiol‐disulfide exchange activity. Substitution of these two residues individually with alanine also abolished hydrogenase activity and strongly reduced ATPase activity, particularly the C72A exchange. Residues in a further conserved GFETT motif were exchanged, but neither hydrogenase enzyme activity nor ATPase activity of the isolated HybG‐HypD complexes was significantly affected. Together, our findings identify a strong correlation between the redox activity of HypD, ATPase activity, and the ability of the complex to mature Hyd enzymes. These results further highlight the important role of thiol residues in the HybG‐HypD scaffold complex during [NiFe]‐cofactor biosynthesis.

## Graphical abstract

We delineate amino acid motifs required for optimal ATPase activity of the HybG‐HypD scaffold complex, which is required for Fe(CN)_2_CO group synthesis during hydrogenase [NiFe]‐cofactor assembly. Substitution of cysteine residues coordinating the 4Fe‐4S cluster or the thiol‐disulfide exchange motif reveals that the intact redox pathway is necessary for optimal ATP‐hydrolyzing activity and functionality of the HybG‐HypD scaffold complex.

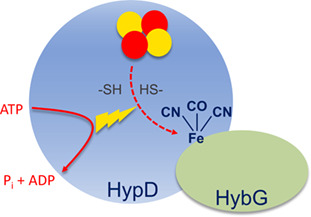

**Abbreviations**
[4Fe‐4S]: iron–sulfur
Hyd: [NiFe]‐hydrogenase

Members of the highly conserved HypD and HypC protein families form the basis of a scaffold complex with which the HypE and HypF proteins are also associated and which is essential for anaerobic synthesis and assembly of the Fe(CN)_2_CO group of the bimetallic NiFe(CN)_2_CO‐cofactor in [NiFe]‐hydrogenases (Hyd). The cofactor is essential for the formation of a catalytically active large subunit of Hyd [1, 2, 3, 4]. The nickel ion is added in a final step by HypA and HypB after prior synthesis and insertion of the Fe(CN)_2_CO group by the scaffold complex into the Hyd apo‐large subunit. The carbonyl and cyano diatomic ligands are made and attached to the iron ion by the combined actions of HypC, HypD, HypE, and HypF, whereby the latter two generate the two cyano ligands from carbamoylphosphate [1, 5, 6, 7]. The metabolic precursor for the anaerobically generated carbonyl ligand is still unresolved [8]; in certain bacteria that synthesize oxygen‐tolerant Hyd, however, CO is made by the decarbonylation of formyl‐CoA, which is generated from formyltetrahydrofolate by the HypX protein [9]. In the absence of oxygen, the HypCD (EF) scaffold complex is likely directly responsible for CO ligand synthesis [1, 2, 3, 4, 10]. The scaffold complex is proposed ultimately to coordinate the Fe(CN)_2_CO moiety of the cofactor via conserved amino acid residues Cys 41 on HypD (*E. coli* numbering) and Cys2 on HypC (Fig. 1), before its transfer into the apo‐large subunit [2, 3, 4, 11, 12].

**Fig. 1:**
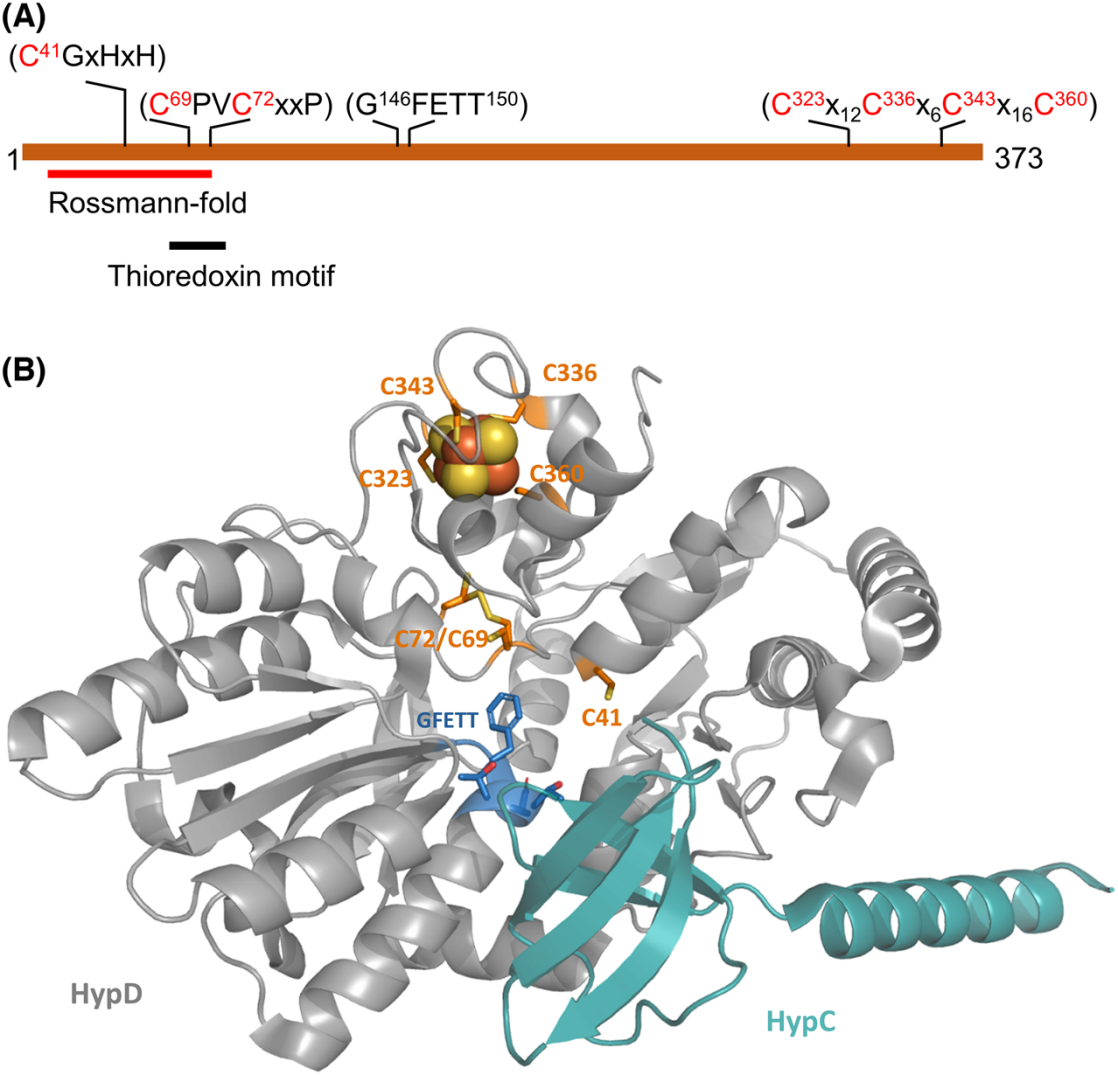
Structural representation of a HypCD scaffold complex. (A) A linear representation of the HypD protein is shown. Highlighted are the important cysteine residues along with the locations of the Rossmann fold, the thioredoxin‐like motif and the GFETT motif [11]. (B) Shown is the crystal structure of the HypC‐HypD scaffold complex from *Thermococcus kodakarensis* (modified from PDB entry 3VYR) [12]. HypD is shown in gray, while HypC is shown in cyan. Conserved cysteine residues are shown in ball‐and‐stick representation (gold), while the residues encompassing the GFETT motif are shown in blue. The [4Fe‐4S] cluster is shown as red and gold spheres. The numbering of amino acid residues is based on *Escherichia coli* HypD: the corresponding residue numbers in HypD from *T. kodakarensis* are C38 (C41), C66 (C69), C69 (C72), C323 (C323), C338 (C336), C345 (C343), and C362 (C360).

A further feature of the HypCD scaffold complex is that HypD coordinates a [4Fe‐4S] cluster and is the only Hyp protein that is redox‐active [1]. One proposal for anaerobic CO ligand synthesis by HypCD is that endogenously produced CO_2_ acts as the metabolic precursor, attached possibly as a formyl group via C2 of HypC [13], and which is suggested to be reductively dehydrated to CO, allowing subsequent attachment to the iron ion bound to C41 of HypD [8, 14]. While the [4Fe‐4S] cluster in HypD has been shown to have a redox potential of −260 mV [15], this would be insufficient to facilitate the reduction of a protein‐ or metal‐bound CO_2_ (formyl‐) to CO, which requires a negative redox potential in the region of −525 mV, under standard conditions [16]. The recent demonstration that HypCD has a low intrinsic ATP‐hydrolyzing activity [17] suggests that the energy released might be potentially harnessed as a conformational activation by the scaffold complex to help overcome this energetic barrier, allowing CO generation [18]. Alternatively, the ATPase activity might conceivably be required for the reductive transfer of the cyano groups from HypE to the HybG‐HypD complex, or for the final transfer of the Fe(CN)_2_CO group from the complex to the hydrogenase large subunit [1, 4].

Structural analyses on HypCD have identified a conserved thiol‐disulfide thioredoxin fold linking the [4Fe‐4S] cluster with C41 in HypD, which would potentially support two electrons and two protons for reductive transfer reactions [4, 11, 12, 15]. This thioredoxin fold contains two conserved cysteine residues (C69 and C72 in *E. coli* HypD; Fig. 1) that are found throughout the HypD family and that are within electron‐transferring distance to C41 [11, 12]. Although HypD does not have a classical ATP‐binding motif, it does have a predicted Rossmann fold, potentially indicative of nucleotide binding, and C69 and C72 form part of this motif [11, 17]. An earlier mutagenesis study demonstrated that when C69 and C72 are exchanged for alanine, the resulting *E. coli* strains synthesizing these variant HypD proteins failed to produce H_2_ gas [14]. The ability of the formate hydrogenlyase complex to produce H_2_ is dependent on the HypCD‐dependent synthesis and insertion of Fe(CN)_2_CO group into the large subunit of Hyd‐3, the hydrogenase component of formate hydrogenlyase complex [1, 19]. Together, these observations highlight the importance of cysteine thiolates in the HypCD scaffold complex and support the role of sulfur chemistry in the maturation of Hyd [1, 14].

In a previous study, we identified an ATPase activity associated with the native HypC‐HypD and HybG‐HypD maturation complexes; however, no potential link to other functions of these complexes, such as its redox activity, was investigated. Therefore, in the current study, we undertook to investigate the potential involvement of the [4Fe‐4S] cluster and the conserved C69 and C72 residues in the ATPase activity of the HybG‐HypD scaffold complex in *E. coli*; HybG is a member of the HypC family of chaperones that is specifically required for the maturation of the H_2_‐oxidizing Hyd‐1 and Hyd‐2 enzymes of *E. coli* [20, 21]. Our current study reveals that both the [4Fe‐4S] cluster and especially the cysteine residues of the thioredoxin fold are important for HypD's ATPase activity.

## Materials and methods

### Bacterial strains, plasmids, and growth conditions

The *E. coli* strains used included MC4100 (F−, *araD139*, ∆(*argF‐lac*)*U169*, λ^−^, *rpsL150*, *relA1*, *deoC1*, *flhD5301*, ∆(*fruK‐yeiR*)*725*(*fruA25*), *rbsR22*, ∆(*fimB‐fimE*)) [22], and its isogenic derivative DHP‐D (Δ*hypD*) [23]. *E. coli* XL1‐Blue (Stratagene (Group), La Jolla, CA, USA) was used for standard cloning procedures. The plasmids used are listed in Table 1. Strains were grown on LB‐agar plates or in LB‐broth at 37 °C [24] for routine microbiological and molecular biological experiments, including cloning. Anaerobic cultivation of strains for hydrogenase enzyme assays and in‐gel enzyme activity staining after native PAGE, or for western blotting experiments, was performed at 37 °C as standing liquid cultures in the buffered rich medium TGYEP (1% w/v tryptone, 0.5% w/v yeast extract, 0.8% w/v glucose, 100 mm potassium phosphate, pH 6.5) [25], supplemented with trace element solution SLA [26]. Cells were harvested anaerobically by centrifugation at 5000 ***g*** for 15 min, at 4 °C, when cultures had reached an optical density at 600 nm (OD_600_) of between 0.8 and 1.2. Cell pellets were generally used immediately for further experiments or were frozen at −20 °C until required.

**Table 1:** Plasmids used in this study.

Plasmid	Characteristics	References or source
pT7‐hypDEF‐hybG	pT7‐7, *hypD*, *hypE*, *hypF*, *hybG* with *C*‐terminal Strep‐TagII, Amp^R^	[27]
pT7‐hypD(C69A)EF‐hybG	Like pT7‐hypDEF‐hybG, but codon 69 in *hypD* TGC to GCT, Amp^R^	This work
pT7‐hypD(C72A)EF‐hybG	Like pT7‐hypDEF‐hybG, but codon 72 in *hypD* TGC to GCC, Amp^R^	This work
pT7‐hypD(G146A)EF‐hybG	Like pT7‐hypDEF‐hybG, but codon 146 in *hypD* GGT to GCT, Amp^R^	This work
pT7‐hypD(F147A)EF‐hybG	Like pT7‐hypDEF‐hybG, but codon 147 in *hypD* TTT to GCT, Amp^R^	This work
pT7‐hypD(E148A)EF‐hybG	Like pT7‐hypDEF‐hybG, but codon 148 in *hypD* GAA to GCA, Amp^R^	This work
pT7‐hypD(T149A)EF‐hybG	Like pT7‐hypDEF‐hybG, but codon 149 in *hypD* ACC to GCC, Amp^R^	This work
pT7‐hypD(T150A)EF‐hybG	Like pT7‐hypDEF‐hybG, but codon 150 in *hypD* ACT to GCT, Amp^R^	This work
pT7‐hypD(C323G)EF‐hybG	Like pT7‐hypDEF‐hybG, but codon 323 in *hypD* TGT to GGT, Amp^R^	This work
pT7‐hypD(C323D)EF‐hybG	Like pT7‐hypDEF‐hybG, but codon 323 in *hypD* TGT to GAT, Amp^R^	This work
pT7‐hypD(C323H)EF‐hybG	Like pT7‐hypDEF‐hybG, but codon 323 in *hypD* TGT to CAT, Amp^R^	This work
pT7‐hypD(C360G)EF‐hybG	Like pT7‐hypDEF‐hybG, but codon 360 in *hypD* TGC to GGT, Amp^R^	This work
pT7‐hypD(C360D)EF‐hybG	Like pT7‐hypDEF‐hybG, but codon 360 in *hypD* TGC to GAC, Amp^R^	This work
pT7‐hypD(C360H)EF‐hybG	Like pT7‐hypDEF‐hybG, but codon 360 in *hypD* TGC to CAC, Amp^R^	This work

For the purification of Strep‐tagged HybG‐HypD scaffold complexes, strains transformed with the appropriate plasmid were cultivated anaerobically at 37 °C as static cultures in modified TB medium (2.4% w/v yeast extract, 1.2% w/v peptone from casein, 0.04% w/v glycerol, 0.4% w/v glucose and 0.003% w/v magnesium sulfate heptahydrate) [10], containing 100 μg mL^−1^ of ampicillin. Cultures were incubated until an OD_600_ of 0.4 was reached and then plasmid‐based gene expression was induced by the addition of 0.1 mm isopropyl β‐d‐1‐thiogalactopyranoside (IPTG). Incubation of the cultures was continued at 30 °C for a further 3 h, and cells were subsequently harvested by centrifugation at 5000 ***g*** for 15 min at 4 °C. Cell pellets were either used immediately or stored at −20 °C until use.

### Plasmid construction

Plasmid pT‐hypDEF‐hybG_Strep_ [27] was used as a template for mutagenesis of the *hypD* gene (Table 1). Mutations were introduced using the PCR‐based mutagenesis kit (Q5^®^ Site‐Directed Mutagenesis Kit; New England Biolabs, Frankfurt, Germany), as described by [17], and the corresponding oligonucleotides used are listed in Table 2.

**Table 2:** Oligonucleotide primers used in this study. Underlined bases indicate the substitution introduced in the *hypD* gene.

Primers	Sequence 5′ → 3′	Comment
HypD_C69A_fw	CGGTCCGGGGGCTCCGGTGTGCG	Substitution of cysteine 69 to alanine (TGC to GCT) in pT7‐hypDEF‐hybG
HypD_C69A_rv	TGGATAAACTCAACGTTTTCCG	Substitution of cysteine 69 to alanine (TGC to GCT) in pT7‐hypDEF‐hybG
HypD_C72A_fw	GTGCCCGGTGGCCGTACTGCCGA	Substitution of cysteine 72 to alanine (TGC to GCC) in pT7‐hypDEF‐hybG
HypD_C72A_rv	CCCGGACCGTGGATAAAC	Substitution of cysteine 72 to alanine (TGC to GCC) in pT7‐hypDEF‐hybG
HypD_G146A_fw	CTTCGGCTTAGCTTTTGAAACCACTATG	Substitution of glycine 146 to alanine (GGT to GCT) in pT7‐hypDEF‐hybG
HypD_G146A_rv	AACACCACTTTGCGGGTT	Substitution of glycine 146 to alanine (GGT to GCT) in pT7‐hypDEF‐hybG
HypD_F147A_fw	CGGCTTAGGTGCTGAAACCACTATG	Substitution of phenylalanine 147 to alanine (TTT to GCT) in pT7‐hypDEF‐hybG
HypD_F147A_rv	AAGAACACCACTTTGCGG	Substitution of phenylalanine 147 to alanine (TTT to GCT) in pT7‐hypDEF‐hybG
HypD_E148A_fw	CTTAGGTTTTGCAACCACTATGC	Substitution of aspartate 148 to alanine (GAA to GCA) in pT7‐hypDEF‐hybG
HypD_E148A_rv	CCGAAGAACACCACTTTG	Substitution of aspartate 148 to alanine (GAA to GCA) in pT7‐hypDEF‐hybG
HypD_T149A_fw	AGGTTTTGAAGCCACTATGCCGA	Substitution of threonine 149 to alanine (ACC to GCC) in pT7‐hypDEF‐hybG
HypD_T149A_rv	AAGCCGAAGAACACCACTTT	Substitution of threonine 149 to alanine (ACC to GCC) in pT7‐hypDEF‐hybG
HypD_T150A_fw	TTTTGAAACCGCTATGCCGACCA	Substitution of threonine 150 to alanine (ACT to GCT) in pT7‐hypDEF‐hybG
HypD_T150A_rv	CCTAAGCCGAAGAACACC	Substitution of threonine 150 to alanine (ACT to GCT) in pT7‐hypDEF‐hybG
HypD_C323G_fw	GCGCGCGCGTGGTGGTGAGGTAT	Substitution of cysteine 323 to glycine (TGT to GGT) in pT7‐7 hypDEF‐hybG
HypD_C323G_rv	GGGTCATCGCAGACCTGCTGC	Substitution of cysteine 323 to glycine (TGT to GGT), to histidine (TGT to CAT) or to aspartate (TGT to GAT) in pT7‐hypDEF‐hybG
HypD_C360G_fw	CGAAGGAGCGGGTGCCGCGTGGT	Substitution of cysteine 360 to glycine (TGC to GGT) in pT7‐hypDEF‐hybG
HypD_C360G_rv	GAGGAAACCATCAGCGCACCAAACG	Substitution of cysteine 360 to glycine (TGC to GGT), to histidine (TGC to CAC) or to aspartate (TGC to GAC) in pT7‐hypDEF‐hybG
HypD_C360H_fw	CGAAGGAGCGCACGCCGCGTGGT	Substitution of cysteine 360 to histidine (TGC to CAC) in pT7‐hypDEF‐hybG
HypD_C360D_fw	CGAAGGAGCGGACGCCGCGTGGT	Substitution of cysteine 360 to aspartate (TGC to GAC) in pT7‐hypDEF‐hybG
HypD_C323H_fw	GCGCGCGCGTCACGGTGAGGTATTAAC	Substitution of cysteine 323 to histidine (TGT to CAT) in pT7‐hypDEF‐hybG
HypD_C323D_fw	GCGCGCGCGTGATGGTGAGGTATTAAC	Substitution of cysteine 323 to aspartate (TGT to GAT) in pT7‐hypDEF‐hybG

### Nondenaturing PAGE and hydrogenase activity staining

Nondenaturing PAGE was performed according to [28]. Aliquots (25–50 μg of protein) of crude extracts were separated using gels that included 7.5% (w/v) polyacrylamide and 0.1% (w/v) Triton X‐100. Before gel application, the crude extracts were incubated with a final concentration of 4% (v/v) Triton X‐100 at 4 °C for 15 min. Visualization of H_2_‐oxidizing activity of Hyd‐1, Hyd‐2, and Hyd‐3 was performed as described previously [29], whereby gels were incubated overnight at 25 °C in an atmosphere of 95% N_2_ : 5% H_2_. Protein concentration was determined as described previously [30]. Experiments were repeated minimally three times using biological replicates, and a representative gel is shown.

### Protein purification

All protein purification steps were carried out in an anaerobic chamber (Coy Laboratories, Grass Lake, MI, USA) and at 4 °C. Amino acid‐exchange variants of HybG_strep_‐HypD scaffold complexes (henceforth HybG‐HypD complexes) were purified from strain DHP‐D (Δ*hypD*) carrying plasmids encoding the cognate mutated *hypD* gene (Table 1). The StrepII‐tag was only present on the HybG chaperone [17, 21] to facilitate complex isolation, and hereafter, the subscript will be omitted when referring to these complexes. Scaffold complexes were initially purified exactly as described previously [21]. After affinity chromatography on Strep‐tactinXT Sepharose (IBA Lifesciences, Göttingen, Germany), the fractions including enriched scaffold complexes were pooled, buffer‐exchanged into 50 mm Tris–HCl pH 8 containing 50 mm NaCl and applied to a Q‐Sepharose Fast Flow column (1 mL) (Cytiva Europe GmbH, Freiburg, Germany). HybG‐HypD complexes were eluted using a NaCl gradient (0.1–1 m in 50 mm Tris–HCl, pH 8). Purified scaffold complexes were again buffer‐exchanged into 50 mm Tris–HCl pH 8 containing 150 mm NaCl and concentrated by use of Vivaspin ultra‐filters (Sartorius AG, Göttingen, Germany) with a 30 kDa cut‐off. Purified proteins were aliquoted into 100 μL pellets that were flash‐frozen in liquid nitrogen and subsequently stored at −80 °C under an N_2_ atmosphere.

### Western blotting

Aliquots of purified scaffold complexes (typically 5 μg protein), or crude extracts (typically 25 μg protein), were analyzed by electrophoresis in 12.5% (w/v) or 15% (w/v) denaturing polyacrylamide SDS/PAGE [31]. After separation, the denatured polypeptides were either visualized by staining with Coomassie Brilliant Blue, or they were transferred onto a nitrocellulose membrane as described previously [32]. After blocking the membrane, polypeptides were identified by challenging with polyclonal antiserum raised specifically against HybG or HypD [21, 33]. Detection was based on chemiluminescence using the Immuno detection kit SuperSignal West Pico PLUS (Thermo Scientific, Brunswick, Germany) and an Amersham Imager 600 (GE Healthcare Bio‐Sciences AB, Solingen, Germany).

### UV–vis spectroscopy

The spectral properties of the purified HybG‐HypD complexes were analyzed in the wavelength range 280–600 nm using a Shimadzu UV‐1900i UV–vis spectrophotometer (Shimadzu Europe GmbH, Duisburg, Germany) and quartz cuvettes with a 1 cm pathlength. The protein concentration used to record the spectra was typically 1 mg·mL^−1^.

### Determination of ATP‐hydrolyzing activity

The ATPase activity of HybG‐HypD complexes was determined by an HPLC‐based assay, exactly as described previously [17].

All data reported in this study were obtained from experiments performed using minimally three biological replicates unless otherwise stated.

### Structural computational methods

A representation of the location of key conserved amino acid motifs within the structure of the HypC‐HypD complex (PDB entry 3VYR) of *Thermococcus kodakarensis* [12] was visualized with PyMOL (The PyMOL Molecular Graphics System, version 2.5; Schrodinger, LLC, New York, USA).

## Results and Discussion

### ATPase activity of the HybG‐HypD complex depends on the cysteines of the thioredoxin fold

**Fig. 2:**
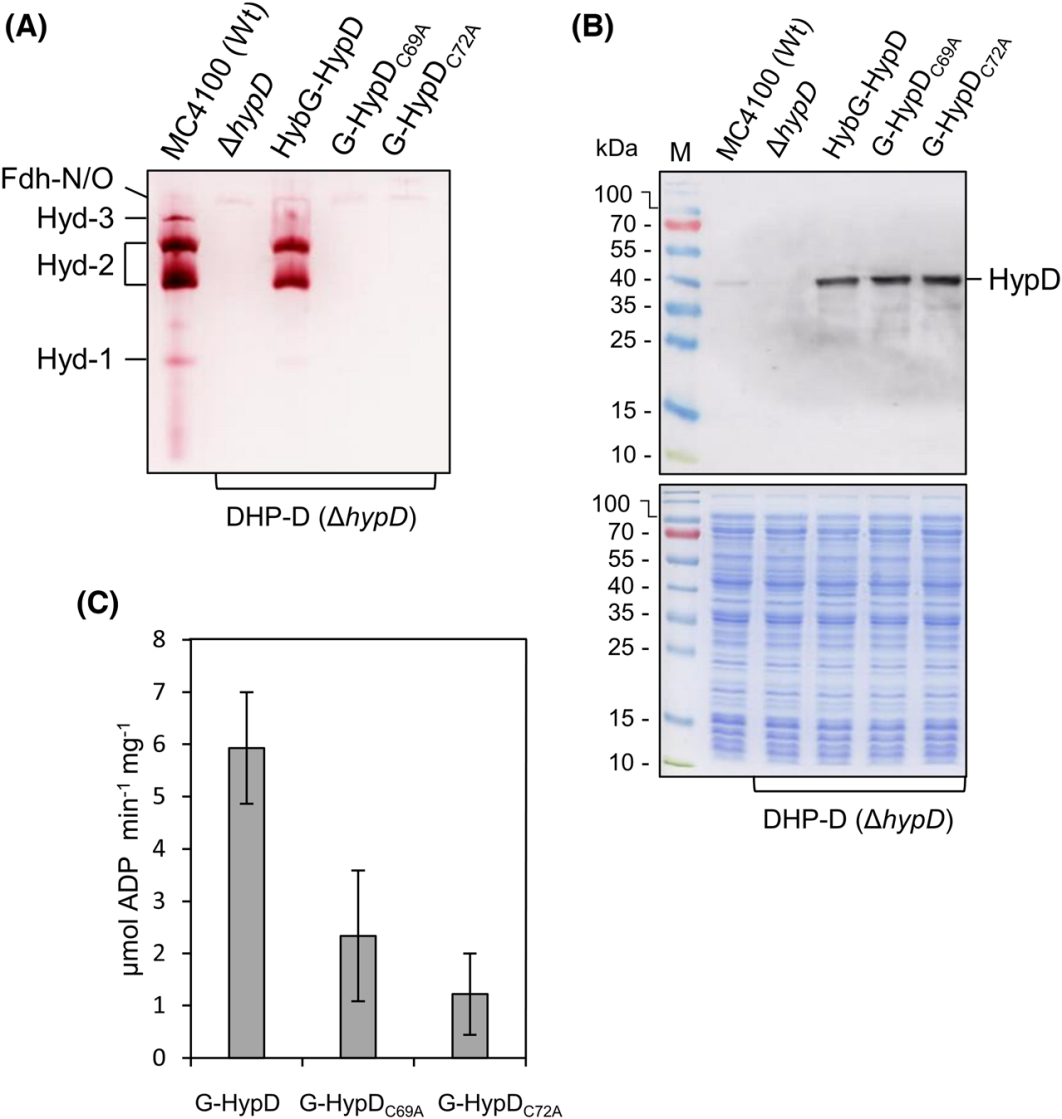
C69A or C72A residue exchanges in HypD abolish maturation capability and impair the ATPase activity of the HybG‐HypD scaffold complex. (A) Shown is a nondenaturing PAGE stained to reveal hydrogenase enzyme activity (see Materials and methods for details). The migrations positions representing Hyd‐1, Hyd‐2, and Hyd‐3 are indicated on the left side of the panel. The weak activity band due to formate dehydrogenases N and O (Fdh‐N/O) [37], acted as a loading control. (B) Polypeptides in aliquots of the same crude extracts shown in part A were separated by SDS/PAGE (12.5% w/v acrylamide) and either analyzed by western blotting using anti‐HypD antiserum (diluted 1 : 4000), or after Coomassie Brilliant Blue staining (lower panel). Molecular mass markers are indicated on the left of each gel, and the migration position of HypD is shown on the right side of the upper panel. (C) ATPase activity of purified native HybG‐HypD complex (G‐HypD) and the indicated variant HybG‐HypD complexes is shown. MC4100, wild‐type (wt); DHP‐D, Δ*hypD*; HypG‐HypD, plasmid‐encoded native Strep‐tagged HybG‐HypD complex; G‐HypD_C69A_, scaffold complex with HypD exchange variant C69A; G‐HypD_C72A_, scaffold complex with HypD exchange variant C72A. Error bars represent standard deviation.

An earlier study reported that H_2_ gas production was abolished in *E. coli* strains synthesizing HypD variants in which the conserved cysteine residues C69 and C72 were substituted with alanine [14]. These cysteine residues form the key structural components of the thiol‐disulfide exchange fold in HypD‐like proteins (Fig. 1A,B) [11, 12]. To determine whether these residues might also be important for the ATPase activity of HybG‐HypD scaffold complexes, we first generated a series of pT7‐hypDEF‐hybG plasmid derivatives carrying mutations in the respective codons 69 and 72 of *hypD* that resulted in HypD proteins bearing an exchange of cysteine for alanine (Table 1). The *hybG* gene on these plasmids carries the coding sequence for a *C*‐terminally located StrepII‐tag, which does not interfere with the ability of the protein to function in the maturation of H_2_‐oxidizing Hyd‐1 or Hyd‐2 enzymes in anaerobically growing *E. coli* (Fig. 2A). Strain DHP‐D (Δ*hypD*) lacks all hydrogenase activity [23] (Fig. 2A), while the introduction of plasmid pT7‐hypDEF‐hybG encoding native HypD restored activity of Hyd‐2 to levels similar in staining intensity to wild‐type strain MC4100; activity of Hyd‐1 was restored to a lesser extent (Fig. 2A). Introduction of plasmids carrying *hypD* genes encoding scaffold complexes bearing either C69A or C72A exchanges in HypD failed to restore any Hyd‐1 or Hyd‐2 enzyme activity, despite the respective HypD proteins being stably synthesized in the strain (Fig. 2B).

Purified HybG‐HypD complexes isolated from the *hypD* mutant DHP‐D (see Materials and methods) were then used to determine whether any impact of the amino acid exchanges on ATPase activity of the complexes had occurred (Fig. 2C). The native HybG‐HypD complex had a specific activity of 5.9 units mg^−1^ (Fig. 2C), which was approximately sixfold higher than the activity determined previously for the HybG‐HypD complex [17]. This improved ATPase specific activity was due to the additional ion‐exchange chromatographic step introduced to the purification procedure (see Materials and methods). SDS/PAGE followed by Coomassie Brilliant Blue staining revealed that an aliquot of the enriched HybG‐HypD complex after Strep‐tag affinity chromatography followed by anion exchange chromatography showed maximally a twofold purification factor when compared to an aliquot of the complex after only Strep‐tag affinity chromatography (Fig. S1). This suggests that after the introduction of the second chromatographic step, the improvement in the specific ATPase activity, when compared to that reported in our earlier study [17], was possibly due to the removal of an inhibitory compound or protein from the complex. Anaerobic purification of the HypC‐HypD complex using the same procedure as that used for HybG‐HypD also resulted in a specific ATPase activity for the complex of 5.79 ± 1.4 units·mg^−1^, which supports this conclusion. The results for complexes carrying residue exchanges in the thioredoxin‐motif revealed that the C69A exchange in HypD reduced the ATPase activity by ~ 64% compared with the activity of the native HybG‐HypD complex, while the C72A exchange reduced activity by over 80% (Fig. 2C).

### HybG‐HypD complexes lacking the [4Fe‐4S] cluster retain partial ATPase activity

**Fig. 3:**
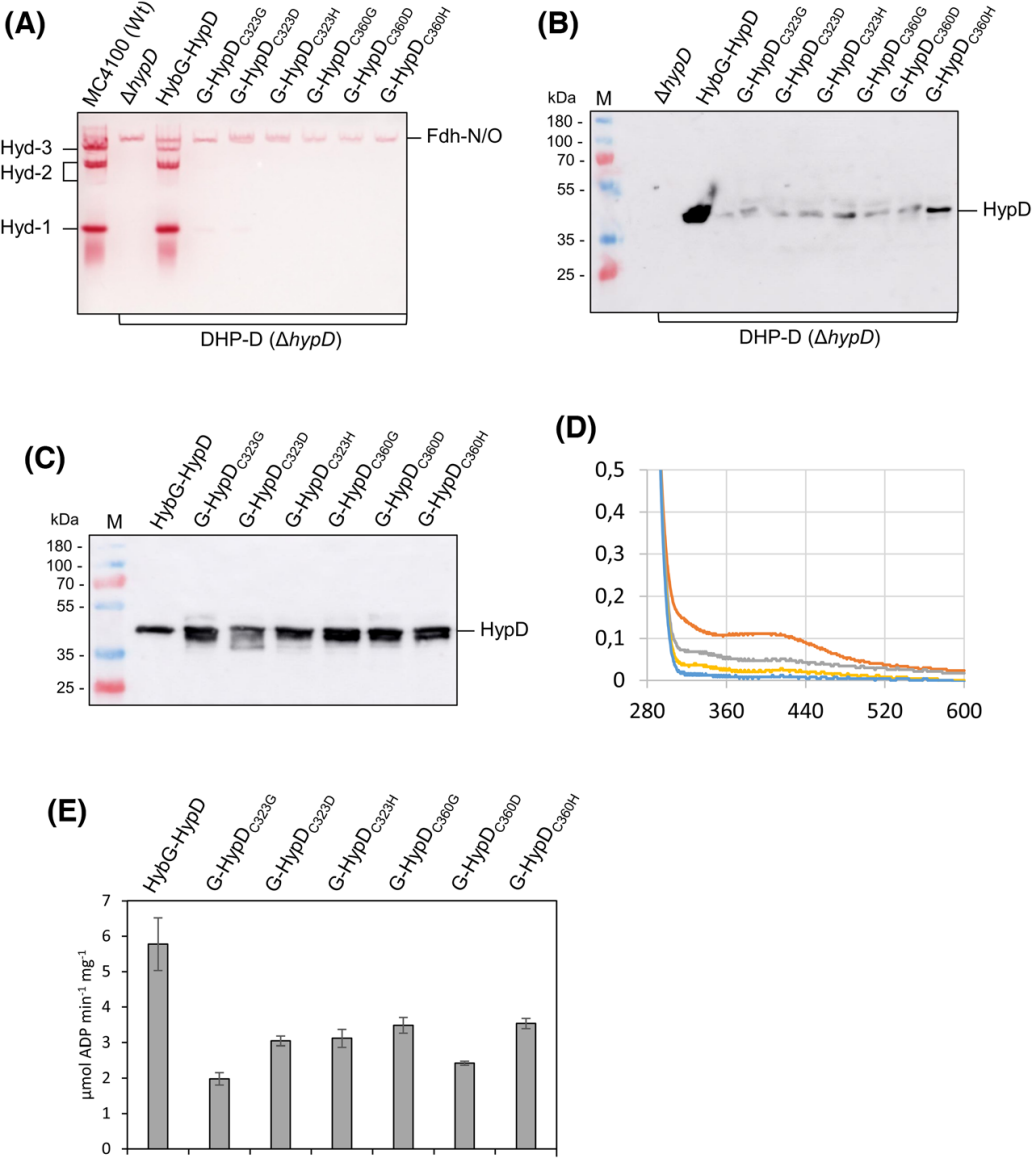
Effects of loss of [4Fe‐4S] cluster on ATPase activity. (A) A nondenaturing PAGE stained to reveal hydrogenase enzyme activity (see Materials and methods for details) is shown. (B) Polypeptides in aliquots of the same crude extracts as in panel A were separated in SDS/PAGE and subjected to western blotting with anti‐HypD antiserum (diluted 1 : 4000). (C) Purified HybG‐HypD complexes (5 μg of protein) were analyzed by western blotting. (D) UV–vis spectra of 20 μM each of selected purified HybG‐HypD complexes were recorded. Native HybG‐HypD (red), HybG‐HypD_C323G_ (gray), HybG‐HypD_C323D_ (yellow), and HybG‐HypD_C323H_ (blue). (E) ATPase activity of purified native HybG‐HypD complexes and the indicated HybG‐HypD [4Fe‐4S] cluster variants is shown. The error bars represent standard deviation.

The four cysteine residues, C323, C336, C343, and C360, that coordinate the [4Fe‐4S] cluster of HypD (Fig. 1), when exchanged for alanine or serine residues, resulted in scaffold complexes that failed to mature Hyd‐3, based on a qualitative H_2_ gas production phenotype [14]. Here, we generated six plasmid derivatives of pT7‐hypDEF‐hybG carrying exchanges in the codons in *hypD* decoding two of these residues, C323 and C360, with codons specifying either glycine, aspartate, or histidine residues (Table 1). These residues were chosen because glycine cannot act as a ligand to coordinate a [4Fe‐4S] cluster, while aspartate and histidine have been shown occasionally to coordinate iron–sulfur clusters [34]. Introduction of the respective plasmids into strain DHP‐D (Δ*hypD*) followed by anaerobic growth and analysis of the crude extracts revealed that the mutated HypD proteins failed to restore hydrogenase activity, based on enzyme activity staining (Fig. 3A). The partial recovery of Hyd‐3 enzyme activity upon introduction of parental plasmid pT7‐hypDEF‐hybG into strain DHP‐D was likely due to maturation of some Hyd‐3 via interaction of overproduced HypD with chromosomally encoded HypC [35]. Analysis of the same crude extracts used to analyze Hyd activity by western blotting with anti‐HypD antiserum indicated that, while detectable, the levels of HypD were considerably lower than the level for native HypD synthesized from pT7‐hypDEF‐hybG (Fig. 3B). This observation is in agreement with data reported previously [14], which was suggested to indicate that mutation of these cysteine residues destabilized the protein due to restricted [4Fe‐4S] occupancy. To address this issue, we isolated all six scaffold complexes with variant HypD proteins, along with the native HybG‐HypD complex and first examined their migration in denaturing PAGE (Fig. 3C). All variant HypD proteins exhibited diffuse migration patterns in western blots with anti‐HypD antiserum when compared to the sharp band exhibited by native HypD. This suggested that these HypD proteins either had retained some secondary structure, even under denaturing conditions, when compared to native HypD, or they exhibited aberrant migration because of the free thiol groups in the remaining free cysteine residues that normally coordinate the [4Fe‐4S]. Moreover, it was noted that, while the anaerobically isolated native HybG‐HypD scaffold complex exhibited a dark brown color, the complexes including the HypD C323 and C360 variants all had a pale straw color. UV–visible spectroscopic analysis of the native HybG‐HypD scaffold complex revealed a broad shoulder around 420 nm (Fig. 3D), characteristic of the presence of a [4Fe‐4S]^2+^ cluster in this protein [36]. By contrast, none of the complexes including HypD variants showed this maximum around 400–420 nm.

Finally, the ATPase activity of each scaffold complex was determined and compared with that of the native HybG‐HypD complex (Fig. 3E). Four of the six variants had an ATPase activity that was ~ 50% lower than that measured for the native scaffold complex, while the C323G and C360D variants had activities that were reduced ~ 60–65% compared with native HybG‐HypD complex (Fig. 3E). Together, these data show that complexes unable to catalyze electron transfer due to impaired [4Fe‐4S] cluster incorporation retained measurable, albeit significantly reduced, ATPase activity compared with the native complex.

### The GFETT motif in HypD is not essential for ATPase activity of the HybG‐HypD heterodimer

**Fig. 4:**
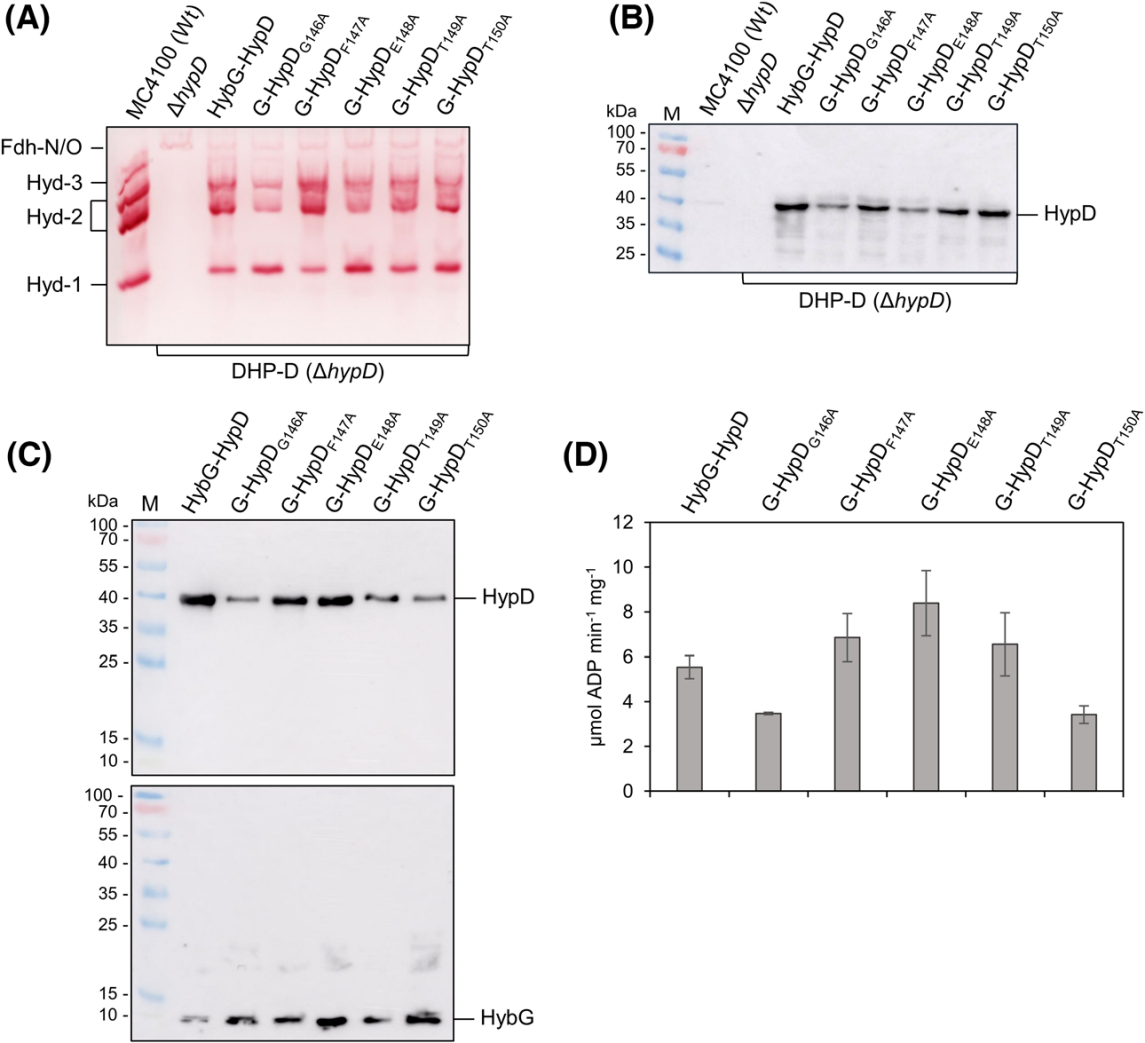
Residue exchanges in the GFETT motif of HypD do not influence ATP‐hydrolyzing activity. (A) Nondenaturing PAGE showing in‐gel hydrogenase activity is shown. (B) Western blot of the same crude extract samples as in panel A was separated in SDS/PAGE and subsequently challenged with anti‐HypD antiserum. (C) Western blots of the indicated purified HybG‐HypD complexes after separation in SDS/PAGE challenged with either anti‐HypD or anti‐HybG antiserum. (D) ATPase activity of purified native HybG‐HypD complexes and the indicated HybG‐HypD GFETT motif variants is shown, and error bars represent standard deviation.

A further conserved motif identified within the HypD family, and located within 7.2 Å of the C69‐C72 motif (Fig. 1), includes the pentapeptide sequence GFETT (residues 146–150 in *E. coli* HypD [11, 14]). This motif is conserved throughout the HypD family [14] and, based on structural analyses, has been suggested to stabilize the thioredoxin fold [11]. Therefore, to determine whether it might be important for the ATPase activity of the scaffold complex, we exchanged each residue within the motif with alanine by creating the corresponding mutant *hypD* alleles on pT7‐hypDEF‐hybG (Table 1). Analysis of strain DHP‐D (Δ*hypD*), transformed with the corresponding plasmids, for Hyd‐1 and Hyd‐2 enzyme activities in crude cell extracts revealed that no residue exchange resulted in complete loss of either Hyd‐1 or Hyd‐2 activity (Fig. 4A). As anticipated, based on these in‐gel activity data, western blotting of these extracts revealed stable synthesis of the HypD variants in crude extracts (Fig. 4B). To determine any consequence of the residue exchanges on the ATPase activity of these variants, the corresponding Strep‐tagged HybG‐HypD scaffold complexes were purified (Fig. 4C) and their respective ATPase activity assayed as described in the Materials and methods. Only two exchanges showed a weak reduction in ATPase activity of ~ 40% compared with the native HybG‐HypD complex, namely exchanges G146A and T150A (Fig. 4D). All other exchanges showed an ATPase activity of the cognate scaffold complex that was at least equivalent to that of the native complex, if not marginally higher.

## Conclusions

The most clear‐cut impact on ATPase activity was shown by a HybG‐HypD scaffold complex bearing a HypD variant in which cysteine 72 was exchanged with an alanine residue. This reduction in ATPase activity correlated with a complete loss of Hyd enzyme activity. C72 functions together with C69 in a thiol‐disulfide‐exchange reaction facilitating electron and proton transfer reactions during ligand generation and transfer [15]. Surprisingly, although the exchange of C69 for alanine caused impaired ATPase activity, the phenotype was less severe than when C72 was exchanged. Currently, we have no explanation for this finding. Nonetheless, this motif is crucial for optimal ATPase activity, which is also in accord with it being part of HypD's predicted nucleotide‐binding Rossmann fold.

Exchanges in the *C*‐terminal cluster of cysteine residues that coordinate the [4Fe‐4S] cluster of HypD also diminished ATPase activity; however, care must be taken in interpreting these results as the stability of HypD in these scaffold complexes was also compromised [14]. Exchanges in the nearby conserved, but functionally unresolved, GFETT motif [14] had little, to no, impact on the ATPase activity of the HybG‐HypD complex, or indeed on the ability of the complex to afford maturation of Hyd‐1 or Hyd‐2. These findings, nevertheless, serve to emphasize further the direct impact of exchanges in the redox pathway from the [4Fe‐4S] cluster to C41, which coordinates the Fe(CN)_2_CO group, on the complex's ATPase activity. This study highlights a biochemical link between redox and sulfur chemistries and the ATPase activity of the scaffold complex. Future studies will determine whether the ATPase activity of the complex is required for the synthesis of the Fe(CN)_2_CO group, or possibly for its final transfer to the [NiFe]‐hydrogenase large subunit.

## Conflict of interest

The authors declare no conflict of interest.

## Author contributions

AH carried out the experiments. AH and RGS designed the experiments and analyzed the data. RGS drafted the manuscript and conceived the study. Both authors read and approved the final manuscript.

## Supporting information


**Fig. S1.** SDS/PAGE analysis of enriched HybG‐HypD complexes.

## Data Availability

The data that support the findings of this study are available in Figs 2, 3, 4 and the Supporting Information of this article.

## References

[1] Böck A , King PW , Blokesch M , Posewitz MC . Maturation of hydrogenases. Adv Microbiol Physiol. 2006;51:1–71. 17091562 10.1016/s0065-2911(06)51001-x

[2] Bürstel I , Siebert E , Winter G , Hummel P , Zebger I , Friedrich B , et al. A universal scaffold for synthesis of the Fe(CN)_2_(CO) moiety of [NiFe] hydrogenase. J Biol Chem. 2012;287:38845–53. 23019332 10.1074/jbc.M112.376947 PMC3493926

[3] Stripp ST , Soboh B , Lindenstrauss U , Braussemann M , Herzberg M , Nies DH , et al. HypD is the scaffold protein for Fe‐(CN)_2_CO cofactor assembly in [NiFe]‐hydrogenase maturation. Biochemistry. 2013;52:3289–96. 23597401 10.1021/bi400302v

[4] Miki K , Atomi H , Watanabe S . Structural insights into [NiFe] hydrogenase maturation by transient complexes between Hyp proteins. Acc Chem Res. 2020;53:875–86. 32227866 10.1021/acs.accounts.0c00022

[5] Paschos A , Bauer A , Zimmermann A , Zehelein E , Böck A . HypF, a carbamoyl phosphate‐converting enzyme involved in [NiFe] hydrogenase maturation. J Biol Chem. 2002;277:49945–51. 12377778 10.1074/jbc.M204601200

[6] Reissmann S , Hochleitner E , Wang H , Paschos A , Lottspeich F , Glass RS , et al. Taming of a poison: biosynthesis of the NiFe‐hydrogenase cyanide ligands. Science. 2003;299:1067–70. 12586941 10.1126/science.1080972

[7] Blokesch M , Paschos A , Bauer A , Reissmann S , Drapal N , Böck A . Analysis of the transcarbamoylation‐dehydration reaction catalyzed by the hydrogenase maturation proteins HypF and HypE. Eur J Biochem. 2004;271:3428–36. 15291820 10.1111/j.1432-1033.2004.04280.x

[8] Roseboom W , Blokesch M , Böck A , Albracht SP . The biosynthetic routes for carbon monoxide and cyanide in the Ni‐Fe active site of hydrogenases are different. FEBS Lett. 2005;579:469–72. 15642360 10.1016/j.febslet.2004.12.013

[9] Schulz A‐C , Frielingsdorf S , Pommerening P , Lauterbach L , Bistoni G , Neese F , et al. Formyltetrahydrofolate decarbonylase synthesizes the active site CO ligand of O_2_‐tolerant [NiFe] hydrogenase. J Am Chem Soc. 2020;142:1457–64. 31830412 10.1021/jacs.9b11506

[10] Soboh B , Stripp ST , Muhr E , Granich C , Braussemann M , Herzberg M , et al. [NiFe]‐hydrogenase maturation: isolation of a HypC‐HypD complex carrying diatomic CO and CN‐ligands. FEBS Lett. 2012;586:3882–7. 23022438 10.1016/j.febslet.2012.09.019

[11] Watanabe S , Matsumi R , Arai T , Atomi H , Imanaka T , Miki K . Crystal structures of [NiFe] hydrogenase maturation proteins HypC, HypD and HypE: insights into cyanation reaction by thiol redox signaling. Mol Cell. 2007;27:29–40. 17612488 10.1016/j.molcel.2007.05.039

[12] Watanabe S , Matsumi R , Atomi H , Imanaka T , Miki K . Crystal structures of the HypCD complex and the HypCDE ternary complex: transient intermediate complexes during [NiFe] hydrogenase maturation. Structure. 2012;20:2124–37. 23123111 10.1016/j.str.2012.09.018

[13] Soboh B , Stripp ST , Bielak C , Lindenstrauß U , Braussemann M , Javaid M , et al. The [NiFe]‐hydrogenase accessory chaperones HypC and HybG of *Escherichia coli* are iron‐ and carbon dioxide‐binding proteins. FEBS Lett. 2013;587:2512–6. 23851071 10.1016/j.febslet.2013.06.055

[14] Blokesch M , Böck A . Properties of the [NiFe]‐hydrogenase maturation protein HypD. FEBS Lett. 2006;580:4065–8. 16814778 10.1016/j.febslet.2006.06.045

[15] Adamson H , Robinson M , Bond PS , Soboh B , Gillow K , Simonov AN , et al. Analysis of HypD disulfide redox chemistry via optimization of Fourier transformed ac voltammetric data. Anal Chem. 2017;89:1565–73. 28029041 10.1021/acs.analchem.6b03589

[16] Thauer RK . Energy metabolism of methanogenic bacteria. Biochim Biophys Acta. 1990;1018:256–9.

[17] Nutschan K , Golbik RP , Sawers RG . The iron‐sulfur‐containing HypC‐HypD scaffold complex of the [NiFe]‐hydrogenase maturation machinery is an ATPase. FEBS Open Bio. 2019;9:2072–9. 10.1002/2211-5463.12743 PMC6886295 31614069

[18] Jeoung J‐H , Dobbek H . ATP‐dependent substrate reduction at an [Fe_8_S_9_] double‐cubane cluster. Proc Natl Acad Sci USA. 2018;115:2994–9. 29507223 10.1073/pnas.1720489115 PMC5866592

[19] Steinhilper R , Höff G , Heider J , Murphy BJ . Structure of the membrane‐bound formate hydrogenlyase complex from *Escherichia coli* . Nat Commun. 2022;13:5395. 10.1038/s41467-022-32831-x 36104349 PMC9474812

[20] Blokesch M , Magalon A , Böck A . Interplay between the specific chaperone‐like proteins HybG and HypC in maturation of hydrogenases 1, 2, and 3 from *Escherichia coli* . J Bacteriol. 2001;183:2817–22. 11292801 10.1128/JB.183.9.2817-2822.2001 PMC99498

[21] Arlt C , Nutschan K , Haase A , Ihling C , Tänzler D , Sinz A , et al. Native mass spectrometry identifies the HybG chaperone as carrier of the Fe(CN)_2_CO group during maturation of *E. coli* [NiFe]‐hydrogenase 2. Sci Rep. 2021;11:24362. 10.1038/s41598-021-03900-w 34934150 PMC8692609

[22] Casadaban MJ . Transposition and fusion of the *lac* genes to selected promoters in *Escherichia coli* using bacteriophage lambda and Mu. J Mol Biol. 1976;104:541–55. 781293 10.1016/0022-2836(76)90119-4

[23] Jacobi A , Rossmann R , Böck A . The *hyp* operon gene products are required for the maturation of catalytically active hydrogenase isoenzymes in *Escherichia coli* . Arch Microbiol. 1992;158:444–51. 1482271 10.1007/BF00276307

[24] Miller J . Experiments in molecular genetics. Cold Spring Harbor: Cold Spring Harbor Laboratory Press; 1972.

[25] Begg Y , Whyte J , Haddock BA . The identification of mutants of *Escherichia coli* deficient in formate dehydrogenase and nitrate reductase activities using dye indicator plates. FEMS Microbiol Lett. 1977;2:47–50.

[26] Hormann K , Andreesen JR . Reductive cleavage of sarcosine and betaine by *Eubacterium acidaminophilum* via enzyme systems different from glycine reductase. Arch Microbiol. 1989;153:50–9.

[27] Soboh B , Lindenstrauss U , Granich C , Javed M , Herzberg M , Thomas C , et al. [NiFe]‐hydrogenase maturation in vitro: analysis of the roles of the HybG and HypD accessory proteins. Biochem J. 2014;464:169–77. 25184670 10.1042/BJ20140485

[28] Ballantine SP , Boxer DH . Nickel‐containing hydrogenase isoenzymes from anaerobically grown *Escherichia coli* K‐12. J Bacteriol. 1985;163:454–9. 3894325 10.1128/jb.163.2.454-459.1985 PMC219143

[29] Pinske C , Jaroschinsky M , Sargent F , Sawers G . Zymographic differentiation of [NiFe]‐hydrogenases 1, 2 and 3 of *Escherichia coli* K‐12. BMC Microbiol. 2012;12:134. 10.1186/1471-2180-12-134 22769583 PMC3431244

[30] Lowry OH , Rosebrough NJ , Farr AL , Randall RJ . Protein measurement with the Folin phenol reagent. J Biol Chem. 1951;193:265–75. 14907713

[31] Laemmli U . Cleavage of structural proteins during the assembly of the head of bacteriophage T4. Nature. 1970;227:680–5. 5432063 10.1038/227680a0

[32] Towbin H , Staehelin T , Gordon J . Electrophoretic transfer of proteins from polyacrylamide gels to nitrocellulose sheets: procedure and some applications. Proc Natl Acad Sci USA. 1979;76:4350–4. 388439 10.1073/pnas.76.9.4350 PMC411572

[33] Pinske C , Jaroschinsky M , Linek S , Kelly CL , Sargent F , Sawers RG . Physiology and bioenergetics of [NiFe]‐hydrogenase 2‐catalyzed H_2_‐consuming and H_2_‐producing reactions in *Escherichia coli* . J Bacteriol. 2015;197:296–306. 25368299 10.1128/JB.02335-14 PMC4272588

[34] Bak DW , Elliott SJ . Alternative FeS cluster ligands: tuning redox potentials and chemistry. Curr Opin Chem Biol. 2014;19:50–8. 24463764 10.1016/j.cbpa.2013.12.015

[35] Haase A , Sawers RG . Exchange of a single amino acid residue in the HybG chaperone allows maturation of all H_2_‐activating [NiFe]‐hydrogenases in *Escherichia coli* . Front Microbiol. 2022;13:872581. 35422773 10.3389/fmicb.2022.872581 PMC9002611

[36] Blokesch M , Albracht SPJ , Matzanke BF , Drapal NM , Jacobi A , Böck A . The complex between hydrogenase‐maturation proteins HypC and HypD is an intermediate in the supply of cyanide to the active site iron of [NiFe]‐hydrogenases. J Mol Biol. 2004;344:155–67. 15504408 10.1016/j.jmb.2004.09.040

[37] Soboh B , Pinske C , Kuhns M , Waclawek M , Ihling C , Trchounian K , et al. The respiratory molybdo‐selenoprotein formate dehydrogenases of *Escherichia coli* have hydrogen: benzyl viologen oxidoreductase activity. BMC Microbiol. 2011;11:173. 10.1186/1471-2180-11-173 21806784 PMC3160892

