# Supplementary material for: A redox‐active HybG‐HypD scaffold complex is required for optimal ATPase activity during [NiFe]‐hydrogenase maturation in Escherichia coli
Source: FEBS Open Bio. 2023 Jan 20;13(2):341–51. doi: 10.1002/2211-5463.13546 (PMC9900092; doi:10.1002/2211-5463.13546)
Supplement: Supplementary file 1 — Fig. S1. SDS/PAGE analysis of enriched HybG‐HypD complexes. [file FEB4-13-341-s001.pdf]

Supplementary Information

A Redox-Active HybG-HypD Scaffold Complex is Required for Optimal ATPase Activity During [NiFe]-Hydrogenase Maturation in *Escherichia coli*

Alexander Haase[1], and R. Gary Sawers[1#]

[1]Institute for Biology/ Microbiology, Martin-Luther University Halle-Wittenberg, Kurt-Mothes-Str. 3, 06120 Halle (Saale), Germany.

[#] Address correspondence to: R.G. Sawers, Institute for Biology/Microbiology, Martin-Luther University Halle-Wittenberg, Kurt-Mothes-Str. 3, 06120 Halle (Saale) Germany; phone +49 345 5526350; Fax. +49 345 5527010; Email gary.sawers@mikrobiologie.uni-halle.de.

Key words: ATPase; cysteine residues; HybG-HypD scaffold; [NiFe]-hydrogenase maturation; thioredoxin-fold

Abbreviations: iron-sulfur, [4Fe-4S]; [NiFe]-hydrogenase, Hyd

Running heading: Determinants of HypD's ATPase activity

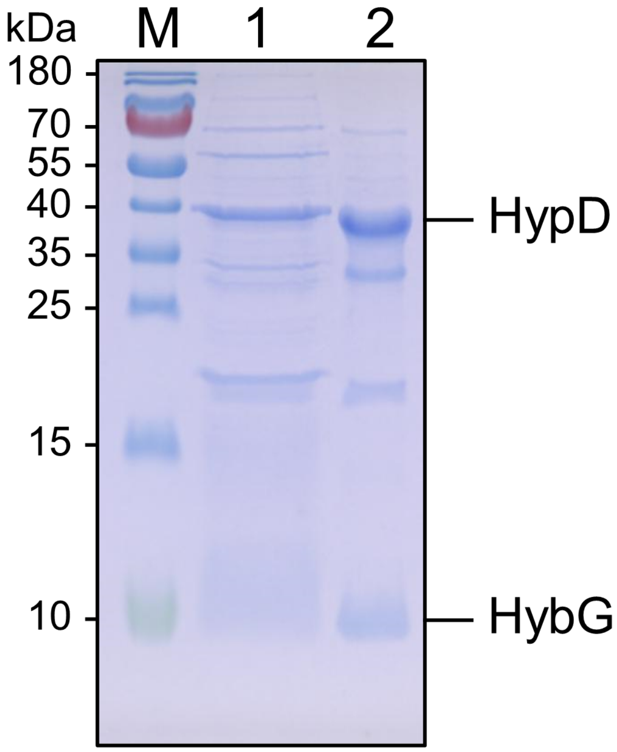

Figure S1. SDS-PAGE analysis of enriched HybG-HypD complexes. Polypeptides in aliquots (5 µg or protein) of samples after Strep-tag affinity chromatography (lane 1) or after Q-Sepharose anion-exchange chromatography (lane 2) are shown. Polypeptides were separated by SDS-PAGE (15 % w/v acrylamide) and stained with Coomassie Brilliant Blue. Molecular mass markers (M) are indicated in kDa on the left of the gel.
